# Interaction and Diffusion Mechanism of Moisture in Power Capacitor Insulating Oil Based on Molecular Simulation

**DOI:** 10.3390/ma17215180

**Published:** 2024-10-24

**Authors:** Changyou Wei, Zhiyi Pang, Rui Qin, Jiwen Huang, Yi Li

**Affiliations:** 1 Faculty of Intelligent Manufacturing, Nanning University, Nanning 530200, China; weichangyou006@163.com (C.W.); pangzhiyi08@163.com (Z.P.); 2 Liuzhou Power Supply Bureau of Guangxi Power Grid Co., Ltd., Liuzhou 545000, China; 18607878410@163.com; 3 School of Automotive Engineering, Liuzhou Polytechnic University, Liuzhou 545000, China; 4 School of Electrical Engineering, Guangxi University, Nanning 530004, China; 5 School of Mechanical and Electrical Engineering, Liuzhou Polytechnic University, Liuzhou 545000, China

**Keywords:** molecular simulation, PXE, molecular simulation, insulating oil

## Abstract

Characterized by its exceptional electrical, physical, and chemical properties, 1-phenyl-1-xylylethane (PXE) insulating oil finds extensive application in the realm of power capacitor insulation. In this study, molecular simulation is employed to investigate the reactivity of PXE insulating oil molecules and the impact of temperature on water diffusion behavior in PXE insulating oil, as well as its solubility. The findings demonstrate a higher propensity for hydrogen atoms in nucleophilic and electrophilic positions within PXE insulating oil molecules to interact with water molecules. The inclusion of a temperature field enhances the Brownian motion of water molecules and improves their diffusion ability within the oil. Furthermore, the temperature field diminishes the interaction force between water molecules and the oil medium. Under the influence of this temperature field, there is an increase in the free volume fraction of PXE insulating oil, leading to a weakening effect on hydrogen bonds between oxygen and hydrogen atoms within PXE insulating oil. Additionally, with increasing temperature, there is an elevation in moisture solubility within insulating oil, resulting in a transition from a suspended state to a dissolved state.

## 1. Introduction

Power capacitors are essential equipment in modern power systems, as they enhance the stability, efficiency, and power quality of such systems through reactive power compensation and load balancing [1,2]. They serve as a crucial component to ensure the secure and cost-effective operation of power systems. The insulation performance of power capacitors plays a crucial role in maintaining voltage stability, reducing reactive power loss, and improving the power factor of the system. The insulating medium, mainly composed of insulating oil or dry insulating material, is utilized to provide effective insulation for capacitors [3]. The insulation performance directly impacts the longevity and safety of the capacitor [4]. The design of a capacitor should be optimized to withstand higher voltages while minimizing electrical energy losses, thereby ensuring the stable operation and energy efficiency of the power system. In practical applications, power capacitors can suffer damage because of various factors [5], such as high voltage, overload, and internal short circuits [6]. Therefore, ensuring the optimal insulation performance of power capacitors is of paramount significance in mitigating these potential issues and guaranteeing the secure operation of the power system [7]. By means of regular inspection and maintenance, potential insulation problems can be promptly detected and resolved, thereby ensuring sustained stability in the operation of power capacitors and safeguarding the safety and efficiency of the power system.

PXE is a synthetic insulating oil that possesses exceptional physical and electrical properties, such as high flash point, low viscosity, high solubility, low pour point, strong coloration ability, and high voltage resistance, facilitates easy purification treatment, and exhibits favorable compatibility with polypropylene film [8,9]. PXE insulating oil is widely utilized in power capacitors, specifically suitable for impregnated film paper composite capacitors and full film capacitors with low field strength. It exhibits a colorless transparent liquid appearance, low density, low acid value, and high flash point. These distinctive characteristics render PXE insulating oil of significant application value within the realm of electrical insulation [10]. The utilization of PXE insulating oil not only enhances the performance of power capacitors [11] but also ensures the stable operation of the power system. PXE insulation oil effectively enhances the insulation performance of power equipment by filling air bubbles in the insulation and preventing the ingress of air or moisture. Additionally, it exhibits a cooling and heat dissipation effect, ensuring the safe operation of electrical equipment through natural or forced circulation for absorbing and dissipating heat. In high-voltage capacitors, PXE insulating oil serves as a medium with unique physical and chemical properties that guarantee its application in high-field strength and large-capacity power capacitors, thereby improving their performance and reliability. However, during operation [12], exposure to temperature variations, oxygen, electric fields, and moisture can induce changes in its physical and chemical properties [13,14]. The issue of moisture aging encountered by insulating oil during operation is a crucial consideration, as it directly impacts the oil’s performance and the safe functioning of equipment [15]. During operation or storage, insulating oil may be contaminated by moisture and impurities, leading to an increase in acid value and sediment content, which alters the properties of the oil. Moisture significantly accelerates the aging process of insulating oil [16], and failure to take timely measures can result in accidents. With the advancement of modern power systems, the significance of power capacitors is progressively increasing, and their failure rate significantly impacts the stable operation of the power system. However, current research primarily focuses on insulating oil for transformers, while studies related to insulating oil for power capacitors are limited to macroscopic experiments, lacking an understanding of its microscopic mechanisms. Therefore, it is imperative to investigate the microscopic mechanism underlying internal moisture diffusion in power capacitor insulating oil.

With the rapid advancement of computer technology, molecular simulation has expanded its applications beyond chemistry to various professional domains [17,18]. Historically, the field of high-voltage insulation primarily relied on macroscopic testing methods for exploration. While these tests partially validate the physical and chemical properties of relevant electrical insulation materials, they fail to provide a comprehensive understanding of the underlying process mechanisms. The application of molecular simulation technology in the field of electrical engineering is continuously expanding, providing a crucial tool and method for investigating microscopic mechanisms [19,20]. By employing a microscopic mixing model with varying concentrations, combined with free volume fraction, mean square displacement (MSD), and diffusion coefficient analysis, the diffusion behavior of moisture in insulating oil can be comprehensively examined. This approach facilitates an enhanced understanding of the relationship between moisture diffusion and the insulating properties of insulating oils. Liao et al. investigated the diffusion behavior of water molecules in oil–paper insulating materials using molecular dynamics simulations [21]. Tian et al. examined the impact of nano-sized SiO_2_ particles on moisture diffusion in naphthene mineral oil through molecular dynamics simulations [22]. Qiu et al. conducted simulations to explore the influence of SiO_2_ nanoparticles on water molecule diffusion within insulating oil at different temperatures [23]. Zhang et al. studied the effect of water on the diffusion behavior of small molecular weight acids in nano-SiO_2_-modified insulating oil [24]. Qiu et al. simulated and compared the diffusion characteristics of water molecules in palm oil and mineral oil under different temperature conditions [25]. Therefore, molecular simulation offers a comprehensive investigation of the diffusion mechanism in insulating oil systems at the microscopic level, making it an invaluable tool for advancing research in this field. Currently, there is a dearth of studies focusing on the micro-level analysis of power capacitor insulating oils. Hence, employing molecular simulation to explore the moisture micro-interaction mechanism in power capacitor insulating oils holds immense significance.

PXE insulating oil is modeled based on molecular simulation in this study. Firstly, the reaction activity of PXE molecules is investigated at the atomic level, followed by an examination of the diffusion behavior of water molecules in PXE insulating oil under different thermal field conditions. The micro diffusion mechanism of water in power capacitor insulating oil is elucidated through the analysis of mean square displacement, diffusion coefficient, and free volume fraction. By combining the radial distribution function (RDF) and interaction analysis, a comprehensive understanding of the relationship between water and insulating oil is achieved, providing theoretical support for understanding the micro-interaction mechanism of water in power capacitor insulating oil.

## 2. Model Construction and Simulation Details

### 2.1. Model Construction

**Figure 1:**
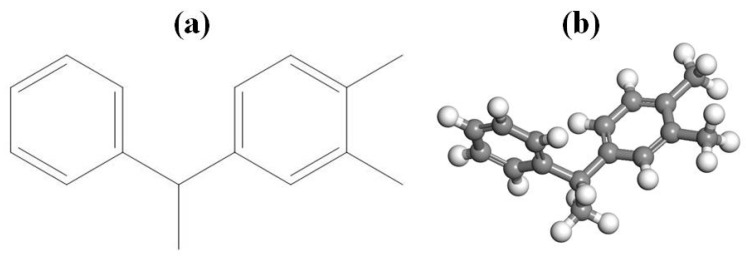
Molecular structure formula of PXE insulating oil (**a**) and molecular model (**b**).

**Figure 2:**
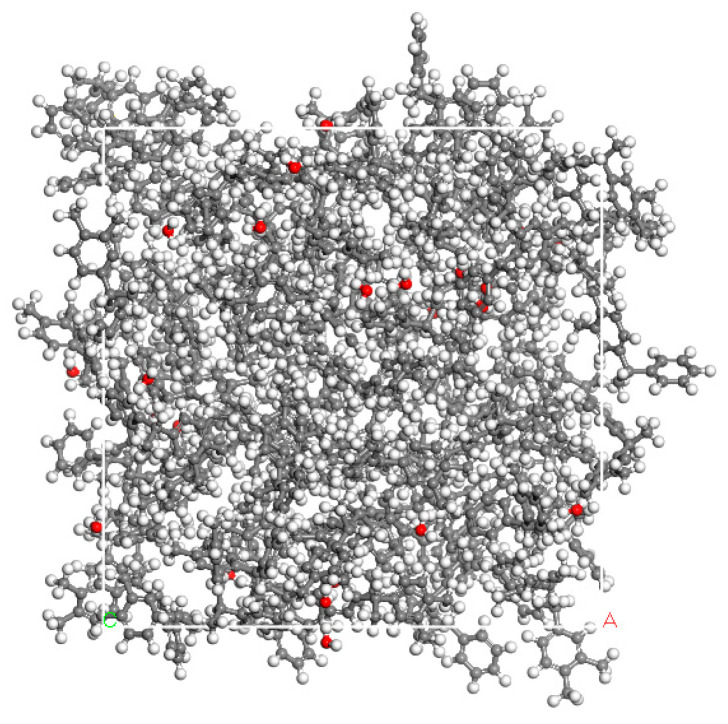
PXE insulating oil mixture model with 2% moisture (White atoms represent hydrogen, grey atoms represent carbon, and red atoms represent oxygen. The letters A and C in the lower right and left corners represent the location of the unit cell, and their colors have no special meaning).

PXE insulating oil is a high-quality dielectric fluid with exceptional insulation properties, primarily utilized in capacitor applications. Its molecular formula is C_14_H_14_, exhibiting stable electrical and thermal performance, low volatility, non-decomposability or explosiveness, high pressure resistance, easy biodegradability, and other desirable characteristics. Consequently, it serves as a hydrocarbon-based insulation oil in numerous countries. Moreover, PXE insulating oil retains sufficient strength even after exposure to high temperatures and remains waterproof, thus aligning with the international development trend as a high-performance product. The molecular structure of PXE is depicted in Figure 1a; the density measures 0.996 g/cm^3^ for this type of insulating oil. The total energy, root mean square (RMS) value, and displacement of the molecular model must satisfy a predetermined convergence tolerance in order for the current state of the molecular model to be deemed reliable. Using the Materials Studio platform and employing the Forcite module, molecular models of PXE were constructed based on the provided molecular structural formula (Figure 1a). Subsequently, geometric optimization was performed to obtain a refined molecular model (Figure 1b). Furthermore, utilizing the Amorphous Cell module, a system comprising 100 PXE molecules was supplemented with 24 water molecules to achieve a mass fraction of 2% water. The resulting model exhibits dimensions of 33.4 Å × 33.4 Å × 33.4 Å and a density of 0.996 g/cm^3^. Subsequent structure optimization and annealing treatment were conducted, leading to the generation of an oil–water mixing model for power capacitor insulation as depicted in Figure 2.

### 2.2. Simulation Details

In the initial stage of molecular simulation, employing the DMol3 module, this investigation employed density functional theory for computational analysis. For the constructed model (Figure 1b), the GGA-BLYP method based on density functional theory was employed to optimize the geometric configuration of PXE molecules and subsequently obtain its stable configuration with the lowest energy. The DMol3 module was then utilized to calculate electrostatic potential, frontier orbitals, and Fukui function in order to determine global and local reaction activity of PXE molecules as well as reactive active sites for both predicted and water molecules.

In the second phase of molecular simulation, utilizing the Forcite module, this investigation employed the COMPASS III force field to conduct calculations. The selection of an appropriate force field is crucial for molecular dynamics simulations and model optimization. COMPASS III, the latest iteration of the COMPASS force field, was specifically developed to offer optimized molecular potentials for atomistic studies, facilitating accurate and simultaneous predictions of a diverse range of molecular properties encompassing structure, conformation, vibration, and thermophysical characteristics. This force field not only encompasses organic molecules but also extends its applicability to inorganic materials, thereby encompassing a broad spectrum ranging from common organic small molecules to polymers, metal ions, metal oxides, and metals. Therefore, the COMPASS III force field was selected for this investigation. Firstly, the micro-water mixing model of PXE insulating oil underwent structure optimization and annealing treatment. The hybrid model was optimized using the Smart method for 500,000 steps to achieve a balanced structure. Cyclic annealing was performed 5 times within the temperature range of 300 to 900 K with an interval of 50 K to obtain a stable state with lower energy. In the dynamics simulation process based on the Forcite module, the NPT ensemble was employed at one atmospheric pressure for a duration of 500 ps. Subsequently, the NVT ensemble was used to simulate temperatures ranging from 303 K to 383 K for another 500 ps. Temperature control was achieved using the Nose method, initial particle velocities were randomly assigned according to Boltzmann distribution, van der Waals interactions were modeled using the Atom-based approach, electrostatic interactions were handled by employing the Ewald method, and an integration step size of 1 fs was set. Kinetic information was collected at intervals of every 500 fs.

## 3. Results and Discussion

### 3.1. Electrostatic Potential Analysis of PXE Insulating Oil

In the study of high-voltage insulating materials, electrostatic potential holds significant importance in comprehending the microscopic characteristics, optimizing electrical properties, and guiding the design of novel materials. By thoroughly investigating the correlation between electrostatic potential and material microstructure, fresh insights and methodologies can be provided for advancing high-voltage technology. The electrostatic potential is determined by both positive nuclear charge and negative electron density, representing the interaction energy between a unit positive charge at a specific point and the current molecular system. A positive electrostatic potential indicates dominance of positively charged nuclear charge, while a negative electrostatic potential signifies contribution from electrons. Within the inner molecular region, because of the proximity to the nucleus, all electrostatic potentials are positive. However, on the surface region of molecules, both electron contribution and nuclear contribution jointly influence, resulting in the appearance of both positive and negative values for the molecular surface’s electrostatic potential owing to the non-uniform distribution of charge density.

**Figure 3:**
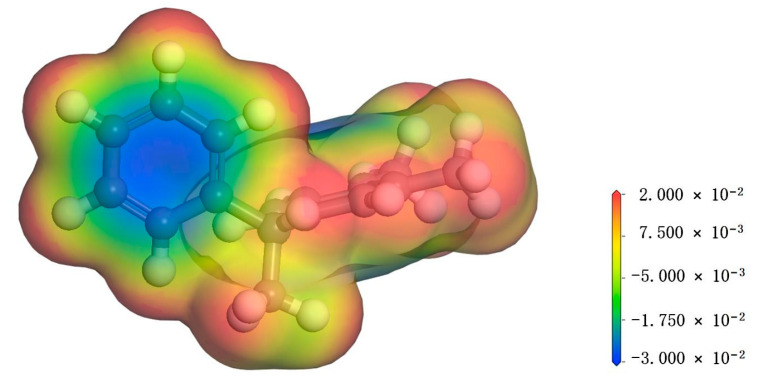
Electrostatic potential of PXE insulating oil molecules.

As depicted in Figure 3, the molecular surface exhibits an electrostatic potential distribution, revealing a positive charge on the hydrogen atom of the PXE molecule (indicated by the red region), which facilitates its affinity towards electron injection. Conversely, the benzene ring (highlighted in blue) displays a negative electrostatic potential, indicating higher electron activity and susceptibility to excitation. Water molecules possess distinctive chemical properties, functioning as both electrophiles capable of accepting protons (H^+^) and nucleophiles that can provide hydroxide ions (OH^−^). This dual nature enables water molecules to serve as electrophilic centers for electron acceptance or nucleophilic centers for electron donation during chemical reactions. Consequently, under appropriate conditions, water molecules can engage in nucleophilic or electrophilic interactions with PXE molecules.

### 3.2. Frontier Orbit and Fukui Function Analysis of PXE Insulating Oil Molecules

**Figure 4:**
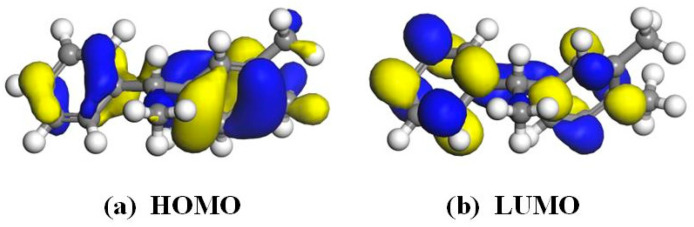
Frontier molecular orbitals of PXE insulating oil (Yellow and blue represent positive and negative orbits, respectively).

**Figure 5:**
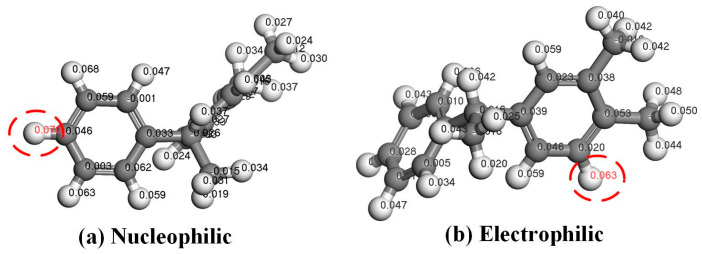
Fukui function of PXE insulating oil molecules (White atoms represent hydrogen, grey atoms represent carbon. The positions marked in red are the most prone to nucleophilic or electrophilic reactions).

According to the frontier orbital theory, the energy of the highest occupied molecular orbital (HOMO) in a molecule is maximized, indicating that electrons are less tightly bound and transitions are more likely to occur. Conversely, the lowest unoccupied molecular orbital (LUMO) possesses minimal energy among all unoccupied orbitals and exhibits high electron affinity. The energy gap (EG) between HOMO and LUMO serves as a measure of electron mobility from occupied to empty orbitals, often employed to assess molecular conductivity. A smaller EG indicates enhanced conductivity and facilitates electron transition from HOMO to LUMO orbitals. In order to identify the specific sites where the PXE molecule exhibits enhanced susceptibility towards nucleophilic and electrophilic reactions, this study exclusively focuses on presenting the HOMO and LUMO orbitals. Figure 4 illustrates the frontier orbital diagram of the PXE molecule, revealing the predominant localization of its HOMO and LUMO orbitals at benzene ring positions. The HOMO orbital energy is −5.227 eV, while the LUMO orbital energy is −0.817 eV, resulting in an EG value of 4.41 eV. Based on this information, further analysis was conducted regarding PXE’s local reactivity using the Fukui function, which indicated that sites with higher *f*+ values are more susceptible to nucleophilic attacks, whereas those with larger *f*− values were prone to electrophilic attacks. Figure 5 demonstrates that the hydrogen atom on the left benzene ring of the PXE molecule possesses the highest *f*+ coefficient, while the hydrogen atom on the right benzene ring has the maximum *f*− coefficient, suggesting their potential interaction with water molecules.

**Figure 6:**
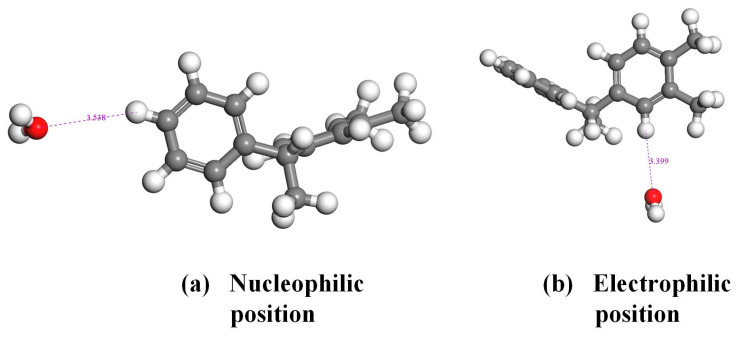
Interaction of PXE insulating oil molecules with water molecules (White atoms represent hydrogen, grey atoms represent carbon, and red atoms represent oxygen).

**Figure 7:**
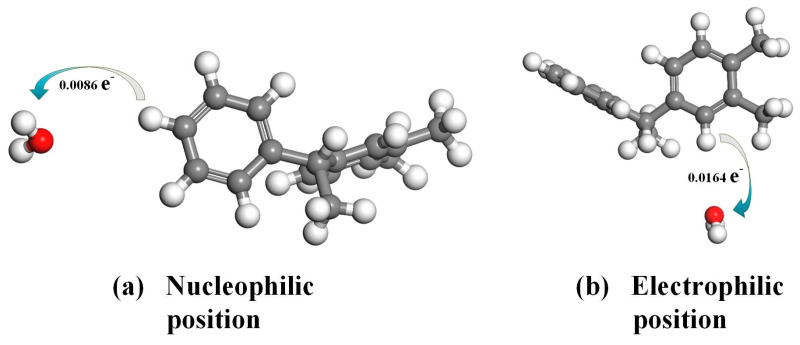
Charge transfer between PXE insulating oil molecules and water molecules (White atoms represent hydrogen, grey atoms represent carbon, and red atoms represent oxygen).

In order to further analyze the interaction between PXE insulating oil molecules and water molecules [26], this study calculated the interaction energy of PXE and water based on the Fukui function’s reaction site and analyzed the extent of charge transfer. The electrostatic potential of 3.1 indicates that the hydrogen atom on the PXE molecule has a positive potential while the oxygen atom on the water molecule has a negative potential; hence, subsequent calculations were performed to investigate their interaction. The two H atoms in the PXE molecule exhibit a higher reactivity towards the water molecule. Additionally, the H atom of the PXE molecule possesses a positive potential, while the O atom of the water molecule carries a negative potential. During their interaction, there is a preferential interaction between the hydrogen atom and the oxygen atom. Therefore, this study employed the DMol3 module to establish a model and placed the oxygen atom of the water molecule at a distance of 5 Å from both hydrogen atoms in Figure 5 to calculate their interaction. Figure 6 illustrates the interaction between the PXE insulating oil molecule and the water molecule. At the nucleophilic position, there is a distance of 3.538 Å between them with an interaction energy of −0.511 kcal/mol. At the electrophilic position, they are separated by a distance of 3.399 Å with an interaction energy of −0.887 kcal/mol. The maximum absolute value for these two positions’ interaction energy lies in the electrophilic position, indicating stronger mutual attractive interactions at this site. The corresponding amount of charge transfer is depicted in Figure 7, where, at the nucleophilic position, PXE transfers 0.0086 e^−^ charges to water, whereas at the electrophilic position, PXE transfers 0.0164 e^−^ charges to water. Based on the above results analysis, it can be concluded that hydrogen atoms in both nucleophilic and electrophilic positions exhibit a higher likelihood of interacting with water molecules.

### 3.3. Diffusion of Moisture in PXE Insulating Oil

The effects of moisture on insulating oil are diverse, encompassing the reduction in breakdown voltage, enhancement in organic acid corrosion ability, acceleration of decomposition and aging of insulating materials, and increase in dielectric loss. Therefore, it is crucial to investigate the mechanism governing moisture movement within power capacitors’ insulating oil at different temperatures. The diffusion motion of water molecules in PXE insulating oil can be characterized by *MSD*, which represents the average distance between particles and their respective starting points at time *t*, as depicted in Equation (1). In Equation (1), ${\vec{r}}_{i}\left(t\right)$ and ${\vec{r}}_{i}\left(0\right)$ represent the position vectors of the particle at time *t* and time 0, respectively. The symbol < > denotes averaging the results within the group. The curve depicting *MSD* against time is fitted to a linear form y = ax + b, with the slope a recorded as an indicator. The diffusion coefficient *D* of water molecules can be obtained from Equation (2). In Equation (2), *N* represents the total number of water molecules in the entire model. Since *MSD* was calculated by averaging all atoms, Equation (2) can be simplified to Equation (3). In Equation (3), *a* corresponds to the slope of the fitting curve.

(1) $$MSD=\langle {\left|{\vec{r}}_{i}\left(t\right)-{\vec{r}}_{i}\left(0\right)\right|}^{2}\rangle$$

(2) $$D=\frac{1}{6N}\underset{n\to \infty }{\lim}\frac{d}{dt}\sum_{i=1}^{N}\langle {\left|{\vec{r}}_{i}\left(t\right)-{\vec{r}}_{i}\left(0\right)\right|}^{2}\rangle$$

(3) $$D=\frac{\left|{\vec{r}}_{i}\left(t\right)-{\vec{r}}_{i}\left(0\right)\right|}{6t}=\frac{a}{6}$$

**Figure 8:**
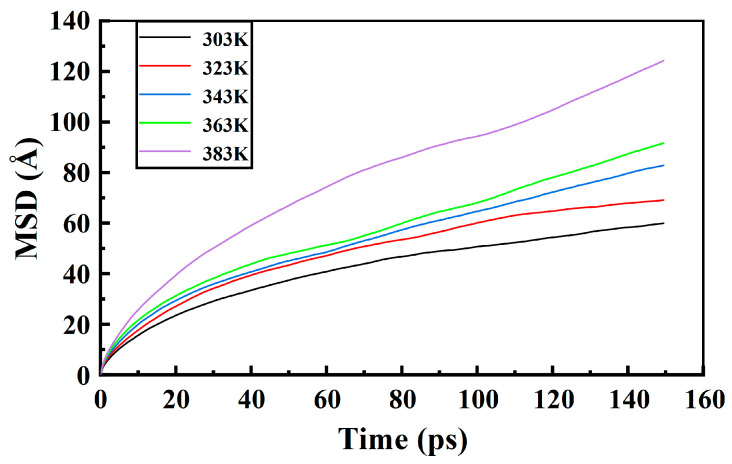
Water molecule MSD of PXE insulating oil at different temperatures.

The *MSD* of moisture in the PXE insulating oil with increasing temperature is illustrated in Figure 8. The time range from 0 to 150 ps within a total duration of 500 ps, as depicted in Figure 8, was selected because of its optimal statistical accuracy for the MSD data analysis. The outcomes obtained through automated fitting using the Forcite module of Materials Studio are presented in Table 1. The results demonstrate that temperature exerts a significant influence on the diffusion behavior of water molecules. As the temperature rises, the motion of water molecules intensifies from slow to fast. In PXE insulating oil containing moisture, the MSD of water molecules ranges from 0 to 100 Å at temperatures between 303 K and 363 K. However, when the temperature reaches 383 K, this range expands to 0–120 Å for water molecules within PXE insulating oil. Table 1 presents the diffusion coefficients of water molecules at different temperatures obtained through mean displacement fitting analysis. With increasing temperature, there is a gradual increase in the MSD of water molecules due to enhanced kinetic energy and intensified irregular motion at higher temperatures. Simultaneously, as temperature increases, the binding between the insulating oil medium and water molecules weakens in the PXE insulating oil, resulting in an increased MSD for these water molecules. The findings of related studies consistently demonstrate that temperature exerts a significant influence on water diffusion in both mineral oil and vegetable oil, aligning with the observed diffusion pattern of synthetic insulating oil in this study [27].

### 3.4. Free Volume of Moisture in PXE Insulating Oil

The measurement of water molecule diffusion motion in insulating oil relies on the assessment of micro-water’s free volume, which holds significant importance. In accordance with Fox and Flory’s free volume theory [28], Equation (4) presents the calculation formula for determining the free volume, where *V_O_* represents the occupied molecular volume and *V_F_* denotes the unoccupied free volume.

(4) $$F_{FV}=\frac{V_{F}}{V_{O}+V_{F}}$$

**Figure 9:**
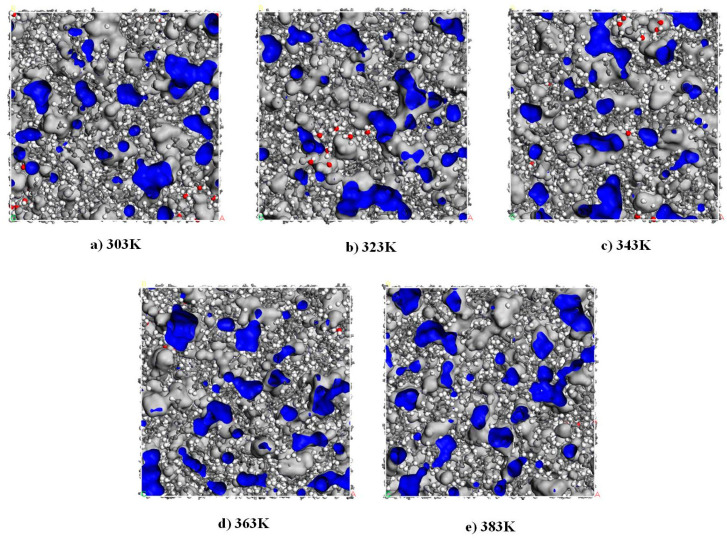
Free volume of PXE insulating oil at different temperatures (The blue part represents the free volume and the gray part represents the occupied volume).

The micro-water free volume in the temperature range of 303 K to 383 K is depicted in Figure 9, with the blue section representing the unoccupied volume and the gray section representing the occupied volume. Upon comparison, it becomes evident that increasing temperature induces changes in both the size and position of micro-water free volume within PXE insulating oil. Moreover, enhanced continuity of the free volume is observed in the model along with a gradual increase in its cross-sectional area. Table 2 presents the free volume data obtained by averaging across these 1000 frames and presents variations in the free volume fraction at different temperatures. As temperature rises, an increment can be noted in the model’s free volume fraction, indicating an intensified impact of elevated temperature on free volume dynamics while reducing binding effects on water molecule movement.

### 3.5. Interaction Energy Between Moisture and PXE Insulating Oil

The interaction energy between moisture and oil molecules plays a crucial role in determining the diffusion behavior of water molecules. Equation (5) can be employed to calculate this interaction energy. In Equation (5), *E*_int_ represents the interaction energy between oil and moisture, *E_Total_* denotes the total potential energy of the entire model, *E_Oil_* signifies the potential energy of insulating oil, and *E_Water_* refers to the potential energy of water. A positive value for the interaction energy indicates repulsion between substances, while a negative value suggests attraction. Moreover, a larger absolute negative value implies stronger binding interactions among these substances.

(5) $$E_{\mathrm{int}}=E_{Total}-E_{Oil}-E_{Water}$$

**Figure 10:**
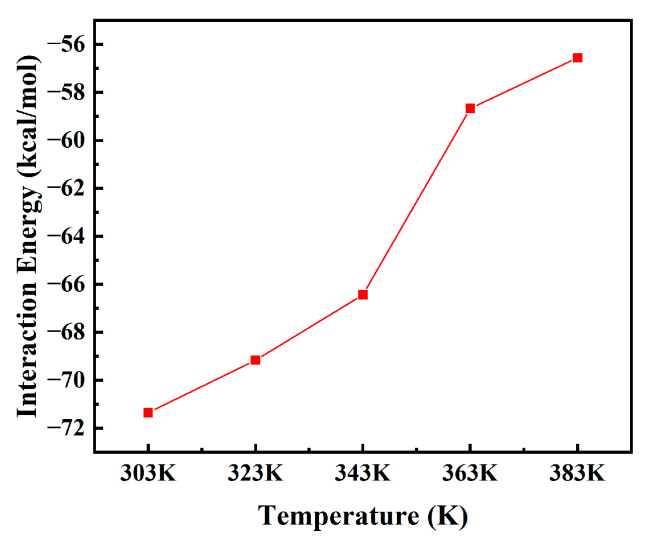
Interaction energy between water and PXE insulating oil at different temperatures.

The variation trend in the interaction energy between moisture and PXE insulating oil in the temperature range of 303 K to 383 K is depicted in Figure 10. At a temperature of 303 K, the PXE insulating oil containing water exhibits the highest level of interaction energy among molecules. As the temperature increases, this interaction energy gradually decreases, indicating a gradual weakening of the binding effect between moisture and PXE insulating oil. The intensified movement of water molecules within the insulating oil can be inferred from the observed change in model interaction energy with increasing temperature.

### 3.6. Radial Distribution Function in PXE Insulating Oil Containing Moisture

**Figure 11:**
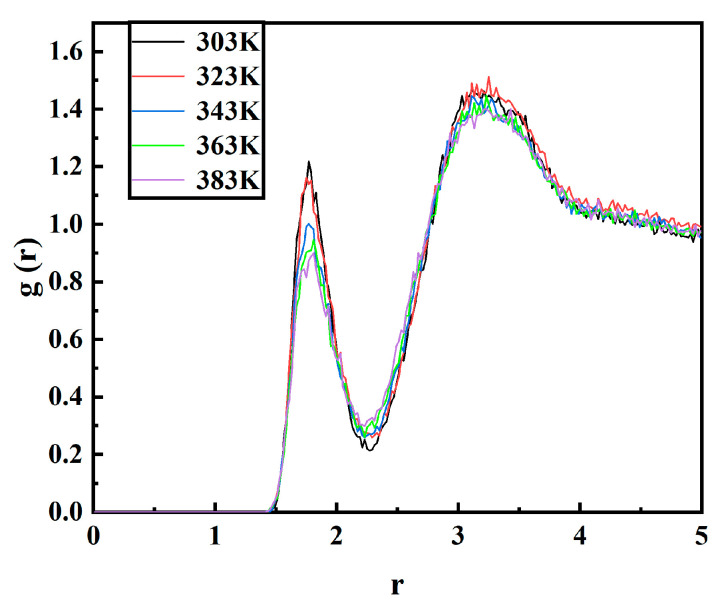
Radial distribution functions of oxygen and hydrogen atoms at different temperatures.

The RDF characterizes the probability distribution of locating a particle at a distance *r* from another labeled particle. Denoted as *g*(*r*), the RDF represents the statistical average of atom arrangement in space and describes the spatial distribution of molecules within a specific system. In an RDF plot, the peak position indicates the most probable interatomic separation distance. Besides investigating structural order, the RDF can also elucidate electron correlation and disclose non-bonded atom interaction modes [29]. Intermolecular forces encompass van der Waals interactions and hydrogen bonding. Figure 11 illustrates the intermolecular RDF for O and H atoms across a temperature range spanning from 303 K to 383 K, where an evident peak emerges at 1.77 Å, signifying intermolecular hydrogen bonding within this interval that diminishes with rising temperature as water molecules progressively dissociate from their initial hydrogen bonds.

### 3.7. Prediction of Moisture Solubility in PXE Insulating Oil

The COSMO-RS method enables the simulation of the system without relying on any experimental data from the desired system. In theory, only one calculation of COSMO is required for each species, resulting in a short computational time and the ability to distinguish isomers. Therefore, COSMO-RS proves to be an effective approach for predicting thermodynamic properties. The solubility of the solute in the solvent can be determined using Equation (6) in COSMO-RS. In Equation (6), *μ_self_* represents the chemical potential of the solute in pure liquid, while *μ_solvent_* denotes its chemical potential in the solvent.

(6) $$\log x=\frac{\mu_{self}-\mu_{solvent}}{RTIn(10)}$$

**Figure 12:**
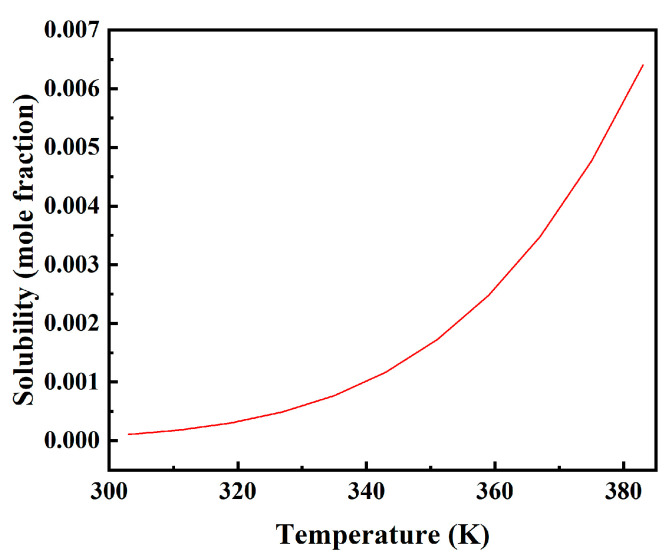
Changes in the solubility of water in PXE insulating oil at different temperatures.

The COSMO calculations of quantum chemistry are conducted for each constituent material, followed by the determination of thermodynamic properties using the COSMO-RS module in AMS 2019.103 software. In the insulating oil, water exists in the following states: dissolved and suspended. When water is in the dissolved state, it has negligible impact on the breakdown voltage of the oil; however, when it is suspended, a significant reduction in breakdown voltage occurs. Figure 12 illustrates the solubility trend of water in power capacitor insulation oil within a temperature range of 303 K to 383 K. As the temperature increases, so does the solubility of water in insulating oil. Consequently, at lower temperatures, water tends to be present as a suspension within insulating oil, leading to weakened diffusion movement of water molecules and a potential detriment to insulation performance.

## 4. Conclusions

In this study, molecular simulation technology is employed to investigate the microscopic interaction behavior of water molecules in PXE power capacitor insulating oil. The atomic-level interaction mechanism is elucidated based on the microscopic characteristics of water molecules in PXE insulating oil, while the molecular-level interaction mechanism is revealed by integrating the diffusion behavior and solubility of water in PXE insulating oil. The following conclusions are derived:

(1) The hydrogen atoms in the PXE insulating oil molecule exhibit a positive electrostatic potential, with the hydrogen atoms located on the surface of the frontier orbital and Fukui function demonstrating a higher propensity for interaction with water molecules compared with other atoms within the PXE molecule. Notably, hydrogen atoms in nucleophilic and electrophilic positions display an increased likelihood of interacting with water molecules.
(2) The diffusion behavior of water in the insulating oil of the PXE power capacitor is influenced by temperature. As the temperature increases, the affinity between the insulating oil medium and water weakens, leading to a gradual increase in mean square displacement and the free volume fraction of water. Consequently, this enhances the diffusion capability of water within PXE insulating oil.
(3) With the increase in temperature, the radial distribution function and interaction energy exhibit a decrease, while the moisture movement intensity within the insulating oil of the PXE power capacitor shows an increase. Consequently, this results in a reduction in the absolute value of the interaction energy between moisture and the insulating oil of the PXE power capacitor, thereby indicating a weakening of their binding effect.

The solubility of water in the insulating oil of PXE power capacitors exhibits an increasing trend with rising temperatures. Comprehensive analysis reveals that elevated temperatures transition the state of water in the insulation oil from a suspended state to a dissolved state, thereby reducing breakdown voltage to some extent. However, temperature elevation also accelerates water diffusion within the insulating oil, leading to the aging of the PXE power capacitor’s insulation oil.

## Figures and Tables

**Table 1:** The diffusion coefficient of water molecules in PXE insulating oil.

Temperature	303 K	323 K	343 K	363 K	383 K
*D* (×10^−4^ cm^2^/s)	0.03172804	0.03862912	0.06193554	0.07656594	0.08961657

**Table 2:** The free volume fraction of water molecules in PXE insulating oil.

Temperature	*V_F_*	*V_O_*	*F_FV_*/%
303 K	6089.42	30,450.09	16.67
323 K	6112.10	30,427.41	16.73
343 K	6228.34	30,311.16	17.05
363 K	6254.16	30,285.35	17.12
383 K	6316.28	30,223.23	17.29

## Data Availability

The raw data supporting the conclusions of this article will be made available by the authors on request.
